# Return to Sport and High-Demanding Activities After Curettage for Benign and Locally Aggressive Bone Tumors

**DOI:** 10.3390/jcm15155953

**Published:** 2026-07-30

**Authors:** Edoardo Ipponi, Lorenzo Andreani, Antonio Scardina, Nicola Lazzaro, Donato Gallone, Francesco Sciacca, Marco Mattei, Antonio D’Arienzo, Paolo Domenico Parchi

**Affiliations:** Department of Orthopedics and Trauma Surgery, University of Pisa, Via Paradisa 2, 56124 Pisa, Italy

**Keywords:** complications, fixation, Functionality, recovery, rehabilitation, quality of life

## Abstract

**Background:** Intralesional curettage is a widely adopted surgical technique in orthopedic oncology. This study aims to evaluate patients’ ability to resume sports and high-demand physical activities after intralesional curettage. Traditional scoring systems, such as the MSTS score, may inadequately capture these performance-related aspects. **Methods:** This study analyzed all patients with benign and locally aggressive bone tumors treated with curettage at our institution between 2016 and 2024. Patients were classified according to histological diagnosis, lesion site, filling material, and the use of plates and screws for stabilization of the treated bone. Major postoperative complications and failures were reported. For each case, data on functional demands and sporting activities were collected using the UCLA Activity Score. UCLA Activity Scale scores were compared at T0, before neoplasm recognition; T1, before surgery; and T2, after surgery at the latest follow-up. **Results:** A total of 138 cases were included. The lower limb was involved in 73 cases and the upper limb in 65 cases. Stabilization with plates and screws was performed in 36 cases. Nine cases (6.5%) experienced postoperative complications. The mean UCLA Activity Scale score was 7.7 at T0, 5.1 at T1, and 7.0 at T2. Overall, 92 of 106 patients (86.8%) who regularly practiced sport before disease onset returned to their main pre-disease sport, although 25 of these 92 patients (27.2%) resumed it at a reduced frequency. Patients requiring internal fixation and those experiencing postoperative complications showed less favorable postoperative activity outcomes. Younger patients were more likely to match or increase their previous activity level. **Conclusions:** The impact of benign bone tumors and their surgical treatment on patients’ activity level is considerable. Curettage can restore limb function and allow a return to sport in most treated patients, especially younger patients, those without complications, and those without the need for internal fixation.

## 1. Introduction

Benign bone tumors are a heterogeneous group of lesions that may differ widely in biological behavior, anatomical distribution, and prognosis. Although these tumors do not usually have metastatic potential, they may affect patients’ clinical conditions. Their local growth, proximity to joints, and ability to weaken the bone may lead to pain and functional limitations. Their growth within the bone can cause a relevant impairment of daily and sport-related activities, and clinical conditions can get even worse in case of pathological fractures. For these reasons, surgical treatment is often required, particularly for symptomatic lesions, those in mechanically critical locations, or those showing radiographic progression.

Intralesional curettage is one of the most widely used surgical strategies for benign and locally aggressive bone tumors. Unlike wide bone resections, curettage aims to preserve native bone stock and native joints while achieving adequate local tumor control. Curettage may be performed alone or combined with local adjuvants–such as phenol, argon beam coagulation, or cryotherapy–to clear the tissue of neoplastic cells further and minimize the risk of local recurrence. After curettage, the resulting bone gap can be filled with different materials, including bone grafts, bone substitutes, or PMMA. In selected cases with large lesions in bony areas under mechanical stress, internal fixation may also be required to stabilize the area and prevent intra-operative or post-operative fractures [1,2,3,4,5]. Recent evidence also supports the use of carbon-fiber-reinforced polyetheretherketone plates, which may provide mechanical stability and facilitate post-operative radiographic surveillance after curettage of large or structurally demanding lesions [4].

Refined over the last decades, this surgical approach can achieve acceptable recurrence rates, relatively few complications, and satisfactory functional outcomes [2,3,4,5,6]. In fact, functional recovery has also been reported as generally favorable after curettage. Several studies suggested that patients treated with intralesional curettage for benign or locally aggressive bone lesions often regain satisfactory limb function and pain relief [1,6,7,8].

However, interpreting functional outcomes in this setting remains challenging. Most studies rely on specific oncological functional scores mainly designed for malignancies and long-term oncological evaluations. In particular, the commonly used Musculoskeletal Tumor Society (MSTS) score was originally developed and validated for assessing global function in severely damaged limbs due to malignant tumors and complex surgical demolitions [3,5,9]. Although the MSTS score is undoubtedly valuable, widely adopted, and useful for comparing outcomes after major oncological procedures, its sensitivity in patients undergoing less invasive treatments such as curettage may be questioned. Indeed, pain relief, improved mobility, and a positive emotional acceptance may be sufficient to achieve relatively high MSTS values. As a consequence, this score may compress functional results toward the upper end of the scale, masking residual limitations that could emerge in more demanding activities.

This limitation is particularly relevant in young and active patients, for whom the goal of treatment is not merely pain control or independent ambulation, but rather the recovery of pre-disease activity levels, participation in high-demand occupational tasks, and even return to sport. The ability to run, jump, tolerate impact, or sustain repetitive mechanical loading may represent a much higher functional threshold than that captured by nowadays’ conventional tumor-specific outcome scores. Nevertheless, evidence regarding return to sport after orthopedic oncological procedures remains scarce, and most available data concern patients treated for malignant bone tumors or major reconstructions rather than patients undergoing curettage for benign or locally aggressive lesions [9,10,11,12].

Therefore, despite the large body of literature supporting curettage as a safe, function-preserving treatment, the true capacity of patients to resume high-demand activities after surgery remains largely unknown. The present study was designed to evaluate the impact of benign or locally aggressive bone tumors, and particularly their surgical treatment by curettage, on patients’ activity levels. Special attention was given to patients’ return to sport and their ability to resume high-demand functional activities, to provide a more specific assessment of post-operative recovery beyond conventional functional scoring systems.

## 2. Materials and Methods

This single-center, retrospective study was conducted in accordance with the ethical standards of the 1964 Declaration of Helsinki and its subsequent amendments [13]. We reviewed all patients with benign or low-grade malignant bone tumors treated with curettage at our institution between January 2016 and December 2024. Preoperative and postoperative activity levels were evaluated.

### 2.1. Inclusion and Exclusion Criteria

Inclusion criteria were: (1) histologically confirmed diagnosis of a primary benign or low-grade malignant bone tumor, including atypical cartilaginous tumors selected for intralesional curettage; (2) surgical treatment with intralesional curettage followed by cavity filling; and (3) availability of preoperative and postoperative clinical and radiological data.

Exclusion criteria were: (1) follow-up shorter than 12 months; (2) association with other open surgical procedures, except for those described in the Surgical Treatment section; and (3) preoperative neurological deficits or systemic diseases that could have impaired tissue healing, prevented adequate rehabilitation, or limited return to high-demand activities.

### 2.2. Surgical Treatment

All procedures were performed under general or regional anesthesia.

**Figure 1:**
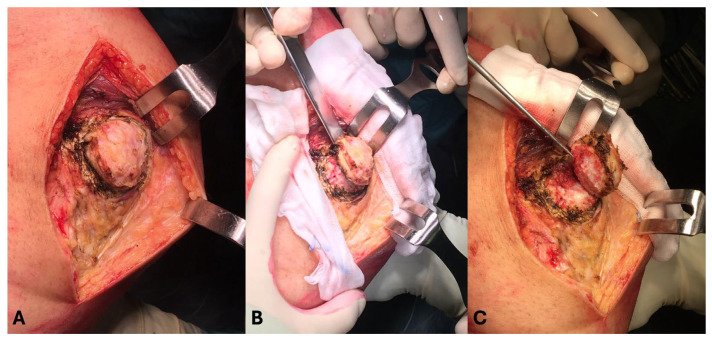
Exposition of the bone and creation of a cortical window (**A**), which is gently mobilized (**B**) to expose the endomedullary lesion (**C**).

The surgical approach, and consequently the patient’s positioning, were chosen based on the lesion’s localization for each patient. Approaches were planned to allow adequate exposure while minimizing soft-tissue damage and preserving future surgical options in case of recurrence. The bone surface is reached and exposed with a blunt dissection (Figure 1A). After fluoroscopic confirmation of the lesion site, a cortical window was created using an oscillating tip saw. Gentle mobilization of the cortical window allows the exposition of the endomedullary lesion (Figure 1B,C).

**Figure 2:**
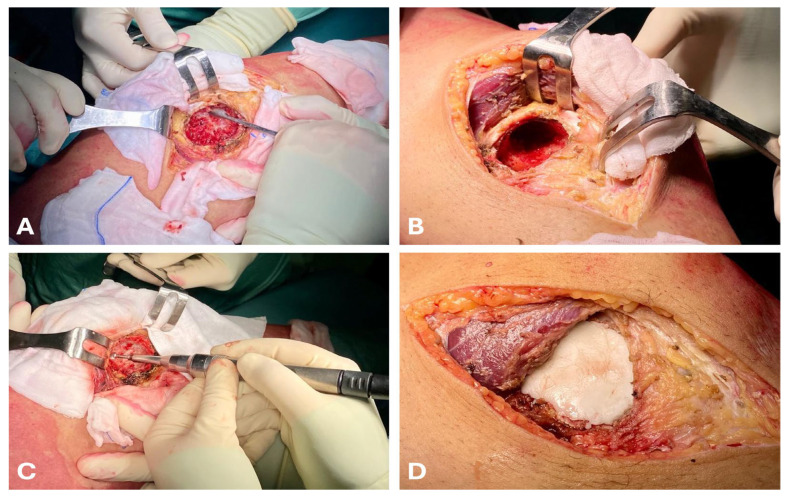
Curettage was carried out with a Volkmann spoon (**A**) until the macroscopically evident neoplasm had been removed (**B**). The surfaces were then treated with a high-speed burr to remove residual tumor cells remaining after the first round of manual curettage (**C**). Finally, the resulting cavity was filled. In the lower-right box, the cavity was filled with PMMA (**D**).

Intralesional curettage was then performed with angled and straight curettes to remove the pathological tissue. The cavity was carefully inspected (Figure 2A), and additional curettage was carried out until macroscopically healthy bone margins were reached (Figure 2B). All the collected material was sent for histopathological examination to confirm the histological diagnosis. A high-speed burr was used to further extend the cavity margins and reduce the risk of residual tumor tissue (Figure 2C). In selected cases, alcoholization or local cryotherapy with cryoprobes was also administered. The resulting bone defect was then irrigated with saline solution and subsequently filled. Filling materials included bone allografts, bone substitutes, or polymethylmethacrylate (PMMA) cement, depending on the size, location, and mechanical requirements of the lesion (Figure 2D). Biological filling was preferred, especially for non-weight-bearing bones and for younger patients, whereas PMMA was mainly reserved for weight-bearing areas, especially in subchondral adult bones.

**Figure 3:**
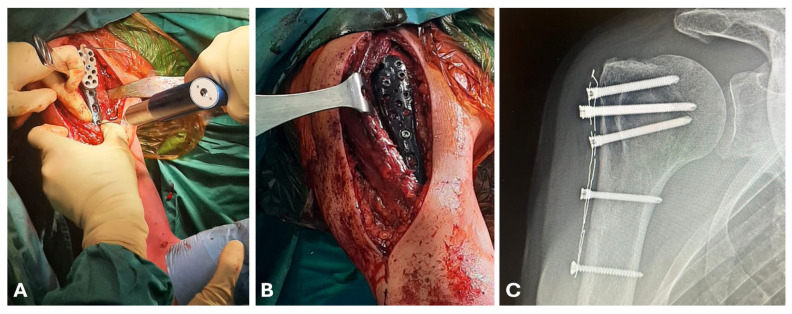
A carbon-PEEK proximal humerus plate is positioned on the bone surface after curettage and bone grafting (**A**). It was finally stabilized with screws (**B**). The final radiological appearance of the plate is shown on the right (**C**).

Internal fixation with carbon-fiber plates and screws was added under fluoroscopic guidance when necessary to restore mechanical stability and prevent impending pathological fractures (Figure 3). After careful hemostasis, wound closure was performed in layers. At the end of the surgery, the closed surgical wound was covered with a flat dressing, and the area was eventually covered with a compression bandage.

A tourniquet was used at the root of the lower limb for lesions located in the distal femur, the patella, fibula, tibia, and foot bones. In the upper limb, arm tourniquets were used for lesions involving the ulna, radius, or hand bones. When used, compression was initiated once the bone surface had been exposed, just before bone curettage, and ended once the cortical window had been set back in place and sealed.

Postoperative management was individualized based on the lesion site, the extent of curettage, and the stability of the bone segment. Weight-bearing and return to physical activity were progressively allowed based on radiographic evidence of healing and clinical recovery.

### 2.3. Post-Operative Management and Follow-Up

All patients, except those with lesions localized to the hand or foot bones, were hospitalized for at least 1 day after surgery and discharged once their local and general conditions were stable.

Rehabilitation started in our institution the day after surgery, and continued after patients’ discharge. The rehabilitative protocol varied depending on the size and location of the treated lesion, the filling material, and the overall stability of the bone, with eventual stabilization also considered. Movement of the nearby articulations and, for the lower limbs, progressive weight-bearing were initiated as soon as possible, in accordance with the principles of early rehabilitation. A gradual return to physical activity was progressively allowed based on radiographic evidence of healing and clinical recovery.

Postoperative follow-up consisted of serial office visits, clinical evaluations, and X-rays performed within 1 month and later at 3, 6, and 12 months after surgery.

### 2.4. Analyzed Data

For each patient who met our inclusion criteria, we recorded their general data, including age and gender. The site, size, and histological diagnosis were recorded for each lesion.

Intra-operative variabilities, including the filling material (bone allograft, bone substitutes, or PMMA) and the eventual use of plates and screws for internal fixation when necessary, were noted.

Before surgery, each patient reported their physical activity level at the time of hospitalization (T1) and at the time of diagnosis and the onset of symptoms (T0). The physical activity level was assessed and classified according to the UCLA Activity Scale, a scoring system ranging from 1 (completely inactive and dependent) to 10 (regular participation in high-impact sports) [14,15]. The same score was used to evaluate each patient’s physical activity level 1 year after surgery. The UCLA score is described in detail in Table 1.

The UCLA activity scale was also used 12 months after surgery or 12 months after the latest surgical treatment in cases of postoperative complications requiring further interventions.

Each complication that occurred during or after surgery, graded II or higher according to the Clavien–Dindo Classification, was reported [16].

### 2.5. Statistical Analysis

Statistical analysis was performed using Stata SE 13 (StataCorp LLC, College Station, TX, USA). Continuous variables were reported as mean and range, while categorical variables were reported as absolute numbers and percentages. Normality was assessed using the Shapiro–Wilk test. Since the UCLA Activity Scale is an ordinal measure and most distributions were not normally distributed, nonparametric tests were used.

Paired comparisons of UCLA scores between different time points were performed using the Wilcoxon signed-rank test. Between-group comparisons, including upper- versus lower-limb lesions, patients with versus without internal fixation, and complicated versus uncomplicated cases, were performed using the Mann–Whitney U test. Fisher’s exact test was used for categorical variables when appropriate. Correlations between age, lesion size, and UCLA score differentials were assessed using Spearman’s rank correlation coefficient.

Multivariable logistic regression was used to evaluate the association between internal fixation and the risk of having a postoperative UCLA score lower than the pre-disease value, adjusting for lesion size, lesion location, histological group, and pre-disease UCLA Activity Scale score. A complementary multivariable linear regression model using the same covariates was performed to evaluate the association between internal fixation and the change in UCLA Activity Scale score from T0 to T2, treated as a continuous outcome. An additional multivariable logistic model was performed with further adjustment for postoperative complications. All tests were two-sided, and statistical significance was set at *p* < 0.05. No formal correction for multiple comparisons was applied.

## 3. Results

One-hundred thirty-eight patients met our inclusion criteria and were included in our study. Our cohort included 61 females (44.2%) and 77 males (55.8%). Their mean age at surgery was 26.8 years, ranging from 12 to 50 years.

### 3.1. Lesion Site and Size

**Figure 4:**
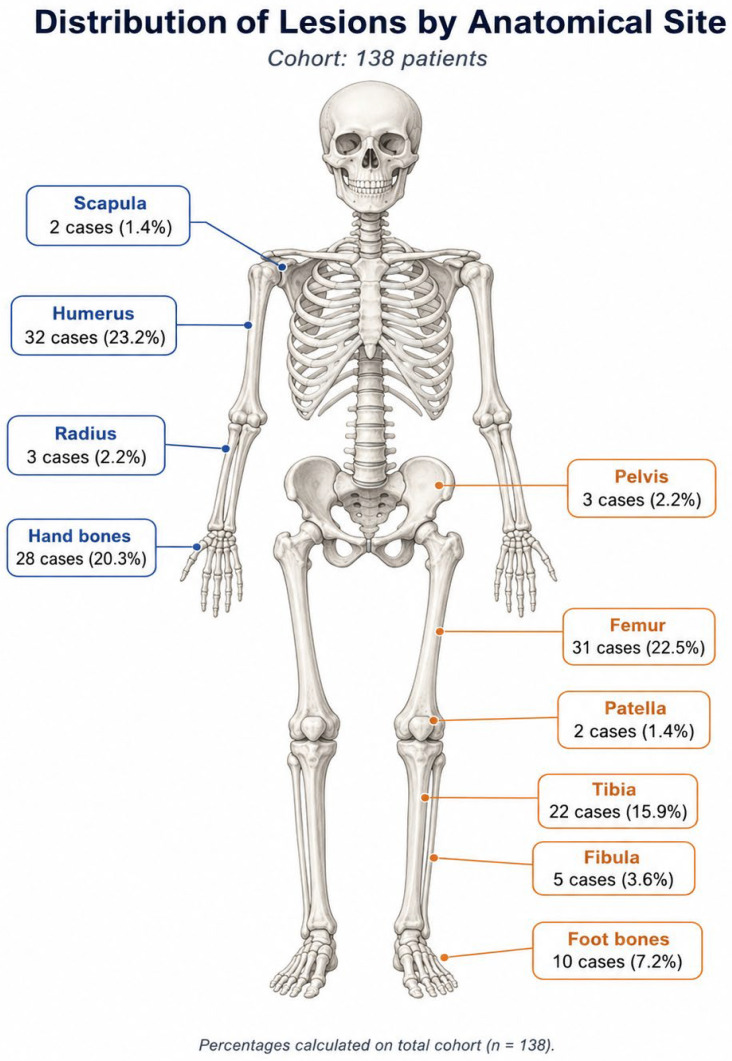
Distribution of lesions by anatomical site. Percentages were calculated on the total cohort of 138 patients.

The lower limb was involved in 73 patients. The pelvis was involved three times. The femur was the most frequent site in the lower limb, hosting the treated lesion in 31 lesions. In two cases, curettage was performed on the patella. Twenty-two patients had their lesions in their tibia and 5 in their fibula. Ten other lesions arose from the foot bones. The remaining 65 patients were treated in their upper limbs. The humerus was the most involved bone of the upper limb, for a total of 32 cases. The remaining cases had their lesions localized to the scapula (2), radius (3), and hand bones (28) (Figure 4).

The mean size of treated lesions was 4.1 (1–12) cm.

### 3.2. Histological Diagnosis

**Figure 5:**
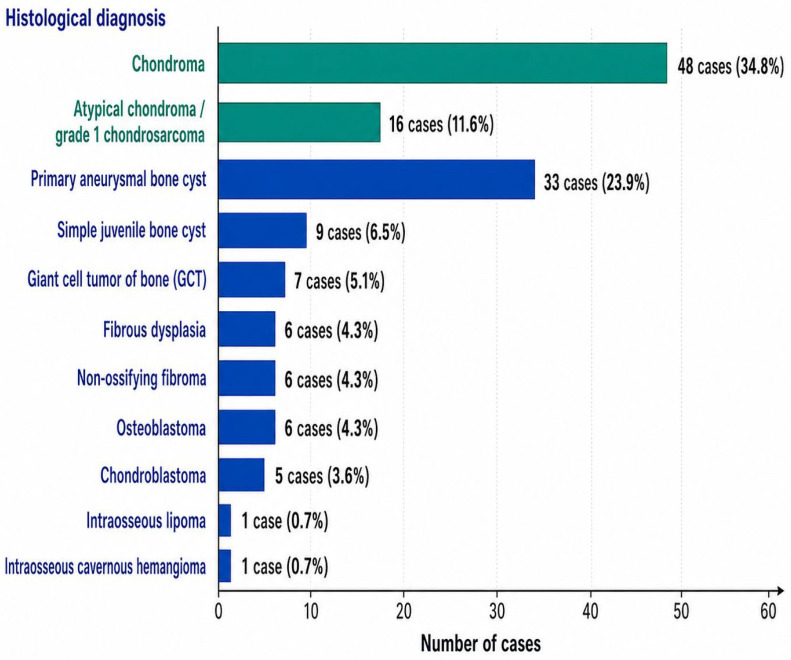
Histological distribution of our cohort. The cartilaginous tumors are pictured in green, the remaining lesions in blue. Percentages were calculated on the total cohort of 138 patients.

Sixty-four cases were diagnosed with cartilaginous tumors: 48 chondromas and 16 atypical chondromas or previously grade 1 chondrosarcomas. These cases were intentionally retained because the present study focused on functional recovery after intralesional curettage. When curettage was selected, the surgical strategy and postoperative rehabilitation pathway were determined primarily by anatomical and mechanical factors. They did not substantially differ according to the final histological classification, which was established on examination of the complete curettage specimen. Thirty-three cases were diagnosed with primary aneurysmal bone cysts, and nine other cases with simple juvenile bone cysts. Seven cases suffered from giant cell tumors of the bone (GCT). Six cases had fibrous dysplasia, and as many had non-ossifying fibroma and osteoblastoma. Five cases had chondroblastoma. Two isolated cases of intraosseous lipoma and intraosseous cavernous hemangioma were also treated (Figure 5).

### 3.3. Filling Material and Internal Fixation

The filling material was bone allografts or bone substitutes in 118 cases (86%). PMMA was used in the remaining 20 cases (14%). Internal fixation was performed in 36 cases (26%), whereas the remaining 102 bones (74%) did not require any further stabilization.

### 3.4. Intra-Operative and Post-Operative Complications and Local Recurrences

Nine of our patients (6.5%) had major complications associated with the surgical treatment. Post-operative fractures represented the most common complication. Fractures occurred in 4 patients. The involved bones were a femur, a humerus, a radius, and a hand phalanx. All cases had been treated with a biological filling and had not received intraoperative stabilization with plates and screws. On average, fractures occurred within 3.2 months (1–5) after surgery. Two cases (femur and radius) required surgical stabilization. The other two cases were treated conservatively.

One case suffered from deep infection, which required further surgical treatment with a new curettage, debridement, intraoperative toilette, and endovenous antibiotic treatment. One case had a large deep hematoma that required surgical drainage. One case with an atypical chondroma of the humerus, treated with curettage and intraoperative stabilization, developed a radial nerve palsy. The associated symptoms disappeared within 6 months after prolonged physiotherapy and neurotrophic therapy. One patient who received internal fixation of the distal femur developed an impingement syndrome that required plate removal as soon as the treated bone healed. Finally, one case of an osteoblastoma of the proximal tibia treated with curettage and PMMA filling had intra-articular leakage, requiring an arthroscopic evaluation to remove the material and seal the cartilage and the subchondral bone discontinuity.

Four cases (2.9%)–two GCTs, one osteoblastoma, and one chondroblastoma–developed local recurrences.

### 3.5. UCLA Activity Scores

**Figure 6:**
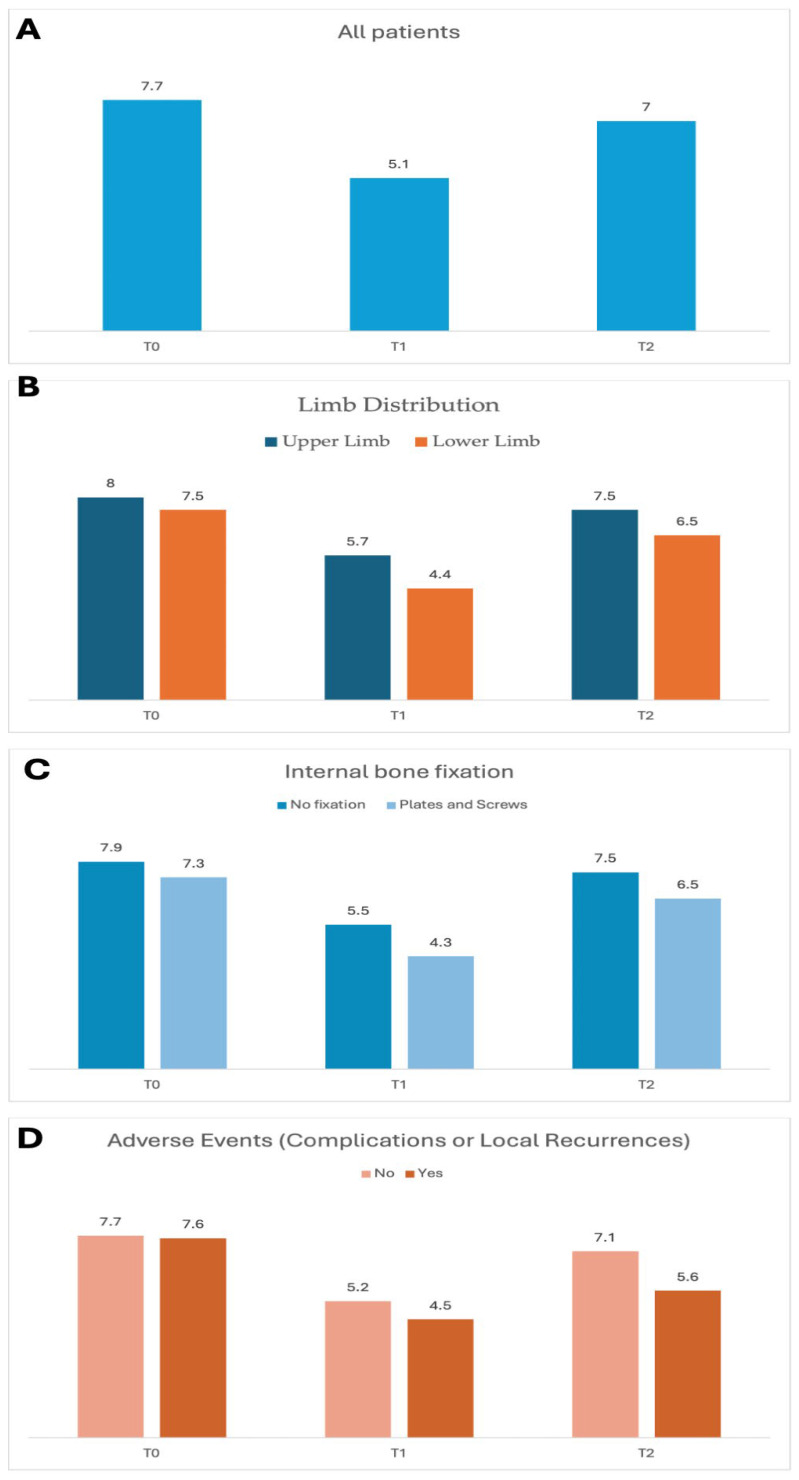
The mean UCLA activity level scores in our population at T0 (before the tumor), T1 (With the tumor and before surgery), and T2 (after surgery). Mean values are reported for the whole cohort (**A**) and dividing the cohort according to lesions’ location (**B**), eventual internal fixations (**C**), and complications (**D**).

The average UCLA activity scores, divided by sub-populations, are reported in Figure 6.

The mean UCLA score at T0 was 7.7 (3–10). Before diagnosis and treatment, 32 patients did not engage in sports on a regular basis, averaging once or more per week. The remaining 106 performed at least one sport regularly. Team sports–including soccer, volleyball, basketball, and rugby–were the most common sports activities in our cohort, as they were the most frequently practiced by 35 of our patients. Twenty-one patients used to go to the gym and perform weight-lifting at least once per week. Swimming was the main sports activity for 12 cases. Ten were runners, and as many were bikers. Tennis was the main sport for 7 of our patients. Four cases performed routine boxing or martial arts. The remaining 7 cases practiced different sports, including rowing, triathlon, calisthenics, and dancing.

At T1, the mean UCLA score decreased to 5.1 (1–10). In 35 cases, the UCLA score at T0 was equal to the one at T1. The remaining 103 cases had at least a mild reduction in their activity levels. On average, between T0 and T1, the UCLA score decreased by 2.7 points (0–8).

At T2, the mean UCLA score was 7.0 (2–10). Within a year after treatment, the mean score was 1.9 (range −6 to 8) points higher than the pre-operative score. The majority of patients (85 cases; 61.6%) achieved higher activity levels than before surgery. In 39 cases (28.2%), the level remained steady. The remaining 14 cases (10.2%) had at least a mild worsening of their UCLA score after surgery compared with their pre-treatment score. The increment of the UCLA score between T1 and T2 was statistically significant according to a two-tailed Wilcoxon signed-rank test (*p* < 0.0001). The UCLA score at T2 was also 0.7 points lower (range: −1 to 7) than at T0. Fifty-two patients (37.7%) had their scores decreased between T0 and T2. This subgroup did not differ from the other analyzed patients in terms of mean UCLA scores at T0 (7.7 for both subgroups) or their age at surgery (26.5 for those who had a worse score and 27.1 for the others; Mann–Whitney U test *p* = 0.7514). Compared to the rest of our cohort, patients whose scores decreased between T0 and T2 had their lesions more frequently localized in the lower limbs (67.9%), as confirmed by Fisher’s exact test (*p* = 0.0082).

In 80 cases (58.0%), the T0 score was equal to the one recorded at T2. Only 6 (4.3%) had better activity levels at T2 than at T0. Although less evident than the difference between T1 and T2, the difference between T0 and T2 was also statistically significant according to a two-tailed Wilcoxon signed-rank test (*p* < 0.0001).

**Figure 7:**
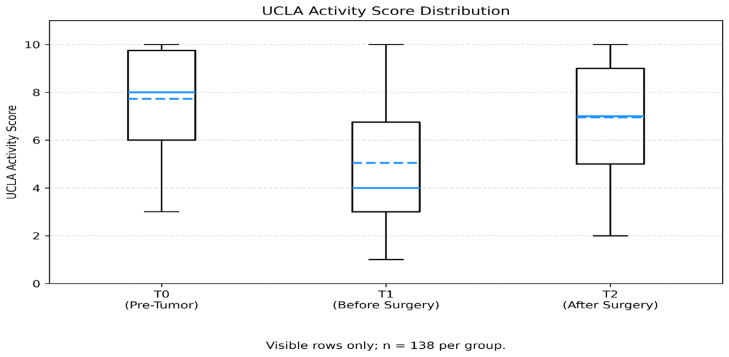
UCLA Activity Score distribution before tumor onset, before surgery, and after surgery. The central horizontal line represents the median, the box indicates the interquartile range, and the whiskers represent the minimum and maximum observed values.

The Box Plot of the UCLA activity scores at T0, T1, and T2 is available in Figure 7.

Patients with upper limb lesions had significantly higher UCLA activity level scores at T2 (7.5) than those with lower limb involvement (6.5), as supported by a Mann–Whitney U test (*p* = 0.0088). The mean UCLA score differential between T1 and T2 was also significantly different, as the activity level recovery was significantly better in the upper limb compared to the lower limb (Mann–Whitney U test: *p* = 0.0207).

An inverse correlation was detected between patients’ age and the difference in UCLA scores between T1 and T2 (Spearman test: Rho= −0.176, *p* = 0.039), suggesting that younger patients had a significantly better recovery than older patients.

Patients who experienced either post-operative complications or local recurrences during their post-operative course had significantly lower T2 scores compared to the others, according to a two-tailed Mann–Whitney U test (*p* = 0.0171).

Patients who required internal stabilization had significantly lower overall UCLA scores at T2 (*p* = 0.0094) and lower T0-T2 differentials (*p* = 0.0092), according to a two-tailed Mann–Whitney U test.

In the multivariable logistic regression model, internal fixation was associated with a higher likelihood of having a postoperative UCLA score below the pre-disease value (adjusted OR 2.80, 95% CI 1.13–6.94; *p* = 0.026). Similar findings were obtained after additionally adjusting for postoperative complications (adjusted OR 2.95, 95% CI 1.18–7.35; *p* = 0.020). In contrast, internal fixation was not significantly associated with the T0–T2 UCLA score change when analyzed as a continuous outcome (adjusted β = −0.30, 95% CI −0.80 to 0.21; *p* = 0.255). Thus, the association between internal fixation and postoperative recovery varied according to the outcome definition.

No correlation was found between lesion size and patients’ return to previous activity levels.

A summary of the main statistically significant outcomes of our cohort is presented in Table 2.

### 3.6. Return to Sport

Among the 106 patients who regularly practiced at least one sport before disease onset, 14 (13.2%) abandoned the same sport they had participated in most. They had an average age of 25.1 years (range 12–50) at surgery. Eight of these 14 used to play team sports. Three used to be runners but could not return to running regularly. The remaining cases could not resume biking, weight-lifting, and playing tennis (one each). The lesions of these 14 patients were localized in the femur (7), humerus (3), scapula (1), patella (1), tibia (1), and fibula (1). Three of them required internal stabilization. Two of them had post-operative complications (deep infection and a fracture), and one had a local recurrence. All these conditions required further surgical treatment. Although these 14 patients who did not return to their sport had a higher incidence of surgical failures, either due to complications or recurrence (21.4%), compared with the rest of our cohort (12.2%), this difference was not statistically significant according to Fisher’s exact test (*p* = 0.3743).

The remaining 92 of 106 patients (86.8%) returned to their main pre-disease sport. Among these 92 patients, 25 (27.2%) resumed their sport at a reduced frequency.

## 4. Discussion

The main finding of the present study is that intralesional curettage with cavity filling allowed most patients with benign or locally aggressive bone tumors to recover a high level of physical activity and return to sport. Although the mean UCLA Activity Scale score did not fully return to the pre-disease value, a clear improvement was observed after surgery compared with the preoperative condition. This suggests that curettage may not only provide local tumor control and pain relief but also restore function relevant to active patients.

The surgical treatment of benign and locally aggressive bone tumors differs substantially from the treatment of primary malignant bone tumors. Malignant lesions often require wide resections, removal of large bone segments, and sacrifice of adjacent soft tissues. In selected cases, vessels, nerves, or joint structures may also be involved in the surgical field. Thereby, even when limb-salvage surgery is an option, these procedures often involve complex reconstructions and lead to long rehabilitation [17,18,19,20,21]. In contrast, curettage for benign bone tumors aims to preserve the natural bone, surrounding joints, and adjacent soft tissues [1]. This less invasive approach may explain the positive functional recovery observed in our cohort. Previous studies have established curettage as a reliable treatment for various benign and locally aggressive bone tumors, with acceptable recurrence rates [1,2,3,4,5,6,7,8,22,23,24]. Additionally, several authors have reported satisfactory functional outcomes, often using scoring systems designed and validated for patients with malignant musculoskeletal tumors [4,5,9].

However, patients with benign bone tumors not only undergo surgeries that are generally less invasive compared to those who are diagnosed with malignancies, but the absence of metastases or chemotherapies does not impact them from a systemic point of view [25]. Furthermore, the psychological and social expectations of patients treated for benign bone tumors are different from those of patients treated for malignant diseases. Patients with sarcoma often face a life-threatening condition and may accept a suboptimal functional result if the limb is preserved and daily activities are possible [25,26,27]. In contrast, patients with benign lesions are usually not exposed to the same oncological risk [28]. For them, the success of treatment is judged not only by local control, but also by the possibility of returning to previous daily activities, including work and sport. This is particularly important as many benign bone tumors occur in young people and adults at the peak of their physical shape and social life, for whom physical activities are a pivotal part of their lives [29].

Our data support this concept. The mean pre-disease UCLA Activity Scale score was 7.7, indicating that many patients regularly performed demanding physical activities before the onset of the tumor. This high baseline value suggests our population may have expectations that go beyond pain relief and independent ambulation.

The decrease in the mean UCLA score from 7.7 before disease recognition to 5.1 before surgery also highlights the clinical burden of bone tumors. Even when histologically benign, these tumors may cause pain, mechanical weakness, fear of fracture, and progressive reduction in physical activity. This impact may be underestimated if only conventional oncological functional scores are used. The MSTS score remains one of the most widely used tools in orthopedic oncology. It was developed to provide a standardized functional evaluation after limb-salvage and ablative procedures for musculoskeletal tumors. It is useful for comparing major oncological reconstructions and for assessing global limb function [9,29,30]. However, its ability to discriminate among high-functioning patients after less invasive procedures may be limited. A patient who walks without aids, has little pain, and accepts the surgical result may achieve a high MSTS score, even if he or she is unable to run, jump, perform impact sports, or return to previous athletic activities. The UCLA Activity Scale may therefore provide complementary information in this setting, as it focuses more directly on patients’ activity level, although its use in this population should be considered exploratory, since the scale has not been specifically validated for patients with bone tumors [14,15].

The improvement observed after surgery confirms the functional effect of curettage. The mean UCLA score increased from 5.1 before surgery to 7.0 after treatment. Most patients improved their activity level after the operation, and 86.8% of those who regularly engaged in sports before disease onset were able to return to their main pre-disease sport. Although most patients returned to their main pre-disease sport, some resumed participation at a lower frequency, highlighting that return to sport does not necessarily imply restoration of the same level of participation. These findings are consistent with the limited data available in the modern literature. Moretti et al. reported that curettage and grafting may relieve athletic-limiting pain and allow full athletic recovery in young athletes with benign lytic bone lesions [12]. In this context, the present study adds specific data on patients treated with curettage rather than major oncological resections, treated with massive allografts or endoprosthetic reconstructions. Nevertheless, these favorable results should not obscure the possibility of persistent functional limitations after surgery. Although curettage allowed most patients to return to sport and high-demand activities, 37.7% of our cohort reported a lower UCLA Activity Scale score at their latest follow-up than before the onset of the disease. For this reason, both surgeons and patients should be aware that curettage may not fully restore pre-disease performance, particularly in young and highly active individuals. Appropriate preoperative counseling is therefore essential to establish realistic expectations, as even a well-performed surgery leading to successful tumor control and satisfactory overall function does not necessarily guarantee a complete return to the patient’s previous level of activity.

Age appeared to be a predominant factor in postoperative recovery. In our cohort, younger patients were more likely to match or improve their previous activity level. This finding is biologically plausible. Younger individuals usually have better bone remodeling capacity, greater muscle mass, and, ultimately, higher physical capacity to adhere to challenging rehabilitation protocols [31,32]. Furthermore, previously active patients at the peak of their physical performance and energy may also have a stronger motivation to resume sports and high-demand activities [33]. This aspect is clinically relevant. In young active patients, more than in any other case, the possibility of returning to sport should be discussed before surgery and taken into account during surgical planning.

Our findings indicate that certain intra-operative or peri-operative events may hinder recovery. The association between internal fixation and postoperative activity was not fully consistent across multivariable outcome definitions: internal fixation was associated with a higher likelihood of a postoperative UCLA score below the pre-disease value in the logistic model, whereas no significant association was observed when UCLA score change was analyzed as a continuous outcome. This discrepancy suggests that the observed association is sensitive to outcome definition and should not be interpreted as evidence of a causal detrimental effect of fixation itself. Rather, internal fixation may reflect greater baseline lesion complexity, mechanically critical locations, and more extensive surgical procedures. While internal fixation is essential when mechanical stability is at risk, these patients may require slower rehabilitation and a more cautious return to impact activities [4,34]. Therefore, patients should be informed that their recovery may take longer than after curettage of smaller or less complex lesions.

Postoperative complications also negatively impacted functional recovery in our population. Issues such as infections, fractures, and other adverse events can impair tissue healing and delay rehabilitation. They may necessitate additional surgery, prolonged immobilization, or extended restrictions on weight-bearing and sports activities. These complications can diminish confidence in the treated limb and prolong the time needed for patients to return to their previous activity levels. Similarly, local recurrence can have a detrimental effect. Recurrence can further compromise bone integrity, cause pain, and require additional treatments. Even when effectively managed, recurrence can also disrupt the rehabilitation process and decrease the likelihood of returning to high-impact activities.

The significant difference between upper and lower-limb lesions was also noteworthy. In our case, upper-limb lesions had a better return to high-demand activities than lower-limb lesions. Lesions localized in the lower limb led to both lower postoperative overall UCLA scores and a lesser capacity to return to sport after the treatment.

Lower-limb lesions, particularly large lesions involving weight-bearing bone segments, are expected to have a greater effect on sport, as running and other impact activities depend heavily on lower-limb loading. Although upper limb function is also essential in many sports and occupations, the impact of surgical treatment on this area might be lower and less invalidating.

We are aware that our study was not free of limitations. The rarity of bone tumors did not allow us to operate on broader populations, and the limited size of our cohort partially limited the statistical significance of some of the data associations we initially sought to investigate. The retrospective nature of our study is another limitation, as it did not allow for the complete standardization of intraoperative treatments and postoperative follow-up procedures for each patient. Retrospective evaluations may also introduce recall bias, particularly regarding pre-disease activity levels. This issue is particularly relevant because the T0 score was used as the reference value for estimating the T0–T2 differential. Patients may overestimate or inaccurately recall their pre-disease activity level, especially when symptoms started several months or years before data collection [35]. Therefore, comparisons involving T0 should be interpreted with caution, as recall bias may have influenced both the apparent magnitude of activity loss before surgery and the degree of recovery after treatment. Conversely, the T1–T2 comparison is less affected by this limitation, as the T1 score was collected during surgical hospitalization. Another limitation lies in the heterogeneity of our population. The cohort included a variety of histological diagnoses, anatomical locations, and filling materials, which may restrict subgroup analysis. The 12-month follow-up period might also be a limitation that could be addressed by conducting similar evaluations after a longer observation period. Finally, the UCLA Activity Scale was originally developed and mainly validated in patients undergoing hip and knee arthroplasty. It has not been specifically validated in orthopedic oncology or bone tumor populations. Therefore, although it was useful to describe changes in self-reported activity level at a group level, our UCLA-based findings should be interpreted as exploratory and may not fully capture the complexity of sport-related recovery after bone tumor surgery. Further studies will also be necessary to compare the outcomes recorded with the UCLA scores with the ones assessed using the MSTS score and confirm differences and the eventual superiority of one score over the other.

Despite these limitations, this study provides valuable and unique insights into how bone tumors and their treatment through curettage affect a patient’s activity level and ability to participate in demanding tasks and sports. These findings are particularly relevant for active patients but have often been overlooked in orthopedic oncology literature. When treating benign bone tumors requiring curettage, orthopedic oncologists should focus not only on local disease control and radiographic healing but also on patients’ functional expectations, including their desire to return to sport and high-demand activities. This aspect is particularly relevant in young and active adults, in whom the impact of surgery may extend beyond pain relief and structural reconstruction. In this perspective, our study may help bring attention to an often-overlooked dimension of orthopedic oncology: the functional and sport-related consequences of curettage for benign bone tumors. Although preliminary, our findings should be considered a first step toward a broader understanding of this topic. Future multicenter studies in larger, more homogeneous populations are needed to better define which outcomes can realistically be expected in high-demand patients and to improve current standards of care.

## 5. Conclusions

Intralesional curettage with cavity filling appears to be an effective function-preserving treatment for benign and locally aggressive bone tumors. It can restore activity levels and allow return to sport in most patients. The best results seem to occur in younger patients, in uncomplicated cases, and in lesions that do not require internal fixation. These findings support the use of activity-based outcome measures in addition to traditional oncological scores. Future prospective studies should include sport-specific endpoints, objective functional testing, and longer follow-up to better define safe timelines and criteria for return to high-demand activities after curettage.

## Figures and Tables

**Table 1:** The UCLA activity scale. The score ranges from 1 to 10, with higher scores indicating greater physical activity.

Score	Activity Level
1	Wholly inactive: dependent on others; cannot leave residence
2	Mostly inactive: very restricted to minimum activities of daily living
3	Sometimes participates in mild activities, such as walking, limited housework, and limited shopping
4	Regularly participates in mild activities
5	Sometimes participates in moderate activities, such as swimming, and can do unlimited housework or shopping
6	Regularly participates in moderate activities
7	Regularly participates in active events, such as bicycling
8	Regularly participates in very active events, such as bowling or golf
9	Sometimes participates in impact sports, such as jogging, tennis, skiing, acrobatics, ballet, heavy labor, or backpacking
10	Regularly participates in impact sports

**Table 2:** A schematic summary of the main statistically significant findings in our cohort.

Comparison	What Was Assessed or Demonstrated	Statistical Test	*p* Value
T1-T2 UCLA score differential	The UCLA score was significantly higher after surgery, compared to the scores before treatment	Two-tailed Wilcoxon signed-rank test	*p* < 0.0001
T0-T2 UCLA score differential	The UCLA score was significantly lower after surgery, compared to the scores before the onset of the tumor	Two-tailed Wilcoxon signed-rank test	*p* = 0.0044
T2 UCLA score in upper-limb vs. lower-limb lesions	Patients with upper-limb lesions had higher postoperative activity scores than patients with lower-limb lesions.	Two-tailed Mann–Whitney U test	*p* = 0.0088
T1-T2 UCLA score differential in upper-limb vs. lower-limb lesions	Patients with upper-limb lesions had greater improvement in their activity level after surgical treatment	Two-tailed Mann–Whitney U test	*p* = 0.0207
Age and T1-T2 UCLA score differential	Younger patients showed better postoperative recovery in terms of activity level after surgery	Spearman correlation test	*p* = 0.039
T2 UCLA score in complicated vs. uncomplicated cases	Patients with complications or recurrence had lower T2 UCLA scores.	Two-tailed Mann–Whitney U test	*p* = 0.0171
T2 UCLA score with vs. without internal fixation	Patients requiring internal fixation had lower T2 UCLA scores.	Two-tailed Mann–Whitney U test	*p* = 0.0094
T0-T2 UCLA score differential with vs. without internal fixation	Patients requiring internal fixation had a worse recovery relative to their pre-disease activity level.	Two-tailed Mann–Whitney U test	*p* = 0.0092

## Data Availability

The data that support the findings of this study are available from the corresponding author upon reasonable request.
